# Fenofibrate decreases the bone quality by down regulating Runx2 in high-fat-diet induced Type 2 diabetes mellitus mouse model

**DOI:** 10.1186/s12944-017-0592-5

**Published:** 2017-10-13

**Authors:** Tianshu Shi, Ke Lu, Siyu Shen, Qiaoli Tang, Kaijia Zhang, Xiaobo Zhu, Yong Shi, Xianglin Liu, Huajian Teng, Chaojun Li, Bin Xue, Qing Jiang

**Affiliations:** 1 Department of Sports Medicine and Adult Reconstructive Surgery, Drum Tower Hospital, School of Medicine, Nanjing University, No. 321 Zhongshan Road, Nanjing, 210008 People’s Republic of China; 2 State Key Laboratory of Pharmaceutical Biotechnology and Jiangsu Key Laboratory of Molecular Medicine and School of Medicine, Nanjing University, No. 22 Hankou Road, Gulou District, Nanjing, Jiangsu Province 210093 China; 3 Joint Research Center for Bone and Joint Disease, Model Animal Research Center (MARC), Nanjing University, Nanjing, 210093 China; 4 State Key Laboratory of Natural Medicines, China Pharmaceutical University, Nanjing, 210009 China; 5 Liver Disease Collaborative Research Platform of Medical School of Nanjing University, Nanjing, 210093 China

**Keywords:** Fenofibrate, Bone quality, Runx2, Type 2 diabetes mellitus

## Abstract

**Background:**

This study is to investigate the effect of fenofibrate on the bone quality of Type 2 diabetes mellitus (T2DM) mouse model.

**Methods:**

T2DM mouse model was induced by high-fat-diet, and the mice were treated with fenofibrate (100 mg/kg) (DIO-FENO) or PBS (DIO-PBS) for 4 weeks. The bone microstructure and biomechanical properties of femora were analyzed by micro-CT and 3-Point bending test. The protein expression was detected by immunohistochemical staining and Western blot. The cell apoptosis was evaluated by TUNEL staining. The Bcl2, caspase 3, and osteoblast marker genes were detected by RT-qPCR.

**Results:**

The biomechanical properties of bones from DIO-FENO group were significantly lower than those in the control and DIO-PBS groups. Besides, the trabecular number was lower than those of the other groups, though the cortical porosity was decreased compared with that of DIO-PBS group because of the increase of apoptotic cells. The expression of osteocalcin and collagen I were decreased after treatment with fenofibrate in T2DM mice. Moreover, the cell viability was decreased after treated with different concentrations of fenofibrate, and the expression of Runx2 decreased after treated with high dose of fenofibrate.

**Conclusion:**

Fenofibrate decreases the bone quality of T2DM mice through decreasing the expression of collagen I and osteocalcin, which may be resulted from the down regulation of Runx2 expression.

## Background

Type 2 diabetes mellitus (T2DM) is a global disease characterized by abnormal metabolism of blood glucose, lipid and protein caused by insulin resistance or abnormal insulin secretion [1, 2]. In addition to increased blood glucose, patients with T2DM also have complications, such as glomerular sclerosis, diabetic foot and brittle fractures [3]. Schwartz et al. found that patients with T2DM have a higher risk of hi p fracture [4]. Bonds et al. reported that bone mineral density (BMD) of T2DM patients increased by 5–10% compared to non-diabetic patients of the same age [5]. However, the pathogenesis of diabetes related bone disease is not clear, and some reports indicated that it may be associated with the increased secretion of advanced glycation end products [6–8], sclerostin [9] and adipokines [10, 11]. On the other hand, it may be related with other complications associated with T2DM, such as retinopathy, which increased the likelihood of falling [12].

The multiple complications of T2DM may affect each other and reduce the survival rate of patients. For example, in about 65% of T2DM patients, who die of cardiovascular disease, dyslipidemia is one of its important predisposing factors [1, 13]. There are a large number of patients with T2DM associated with varying degrees of dyslipidemia characterized by triglyceride (TG) increase and high density lipoprotein decrease [13, 14]. Bijelic et al. reported that the LDL cholesterol and triglycerides were significant risk factors for osteoporosis [15]. Yamaguchi et al. also reported that plasma HDL-Cholesterol levels were significantly and positively correlated with the absolute values of BMD [16]. This result may be related to the development of an inflammatory microenvironment that affects the differentiation and function of osteoblasts caused by the decrease of HDL-Cholesterol.

Fibrates are a class of drugs that can stimulate lipoprotein lipase (LPL) activity by lipolysis of TG in lipoproteins [13], and fenofibrate is one of the most commonly prescribed fibrates. The Fenofibrate Intervention and Event Lowering in Diabetes (FIELD) study showed that fenofibrate can effectively reduce blood TG levels and reduce the risk of total cardiovascular events [17]. And the use of fenofebrate is associated with a decrease in the rate of diabetic amputation and a reduction in retinal lesions [18, 19]. Lee et al. found that gout patients taking fenofibrate may increase the risk of kidney stones [20]. However, there is no report about whether fenofibreate will have an impact on the skeletal system in the treatment of diabetic hyperlipidemia.

In this study, we established a high-fat-diet (HFD) induced T2DM mouse model with dyslipidemia, and evaluated the effects of fenofibrate on bone mass and abnormality in bone microstructure and function. The potential mechanism underlying the effects of fenofibrate was also investigated.

## Methods

### Animals

Two-week-old diet-induced obese (DIO)-C57/BL6 mice were provided by Nanjing Biomedical Research Institute of Nanjing University (NBRI, Nanjing, China). They were kept at room temperature (20–25 °C) with a constant humidity (55 ± 5%) and free access to food and water in a 12/12 h light/dark cycle. All animal experiments were conducted in accordance with the Institutional Animal Ethics Committee and Animal Care Guidelines for the Care and Use of Laboratory Animals of Nanjing University.

### Establishment of T2DM model

Feeding C57BL/6 J mice with HFD is a well-characterized model that results in hyperglycaemia, hyperinsulinemia, insulin resistance, defective islet compensation, and impaired glucose tolerance. For HFD groups (*n* = 12), mice were fed with a HFD (D12492, research diets, New Brunswick, NJ) containing 58.0% fat, 16.4% protein, 25.6% carbohydrates for 10 weeks.

### Animal grouping and treatment

After 10 weeks of feeding with HFD, some mice (*n* = 6) in the HFD groups were gavaged with fenofibrate (100 mg/kg, DIO-FENO) (Sigma, Germany) for 4 weeks [21], and other mice (*n* = 6 were treated with PBS (DIO-PBS). However, for the control group (*n* = 6), routine diet (RD) was provided. At the end of the feeding period, mice were anesthetized with halothane. Tissues were harvested for analysis as described below.

### Microcomputed tomography (micro-CT) analysis

The left femora dissected from the three groups were fixed with 4% paraformaldehyde for 24 h, washed by PBS, and then scanned by a micro-CT scanner (SkyScan, Aarselaar, Belgium). X-ray voltage and current were set at 80 kV and 80 μA, respectively, with a resolution of 18 μm per pixel. Cross-sectional images of femur were used for 3-dimensional histomorphometric analysis of trabecular bone. For the distal femur, the region of interest (ROI) selected for analysis was 5% of femoral length from 0.05 mm below the growth plate to determine trabecular bone mineral density (Tb.BMD), trabecular bone volume per tissue volume (BV/TV), trabecular number (Tb. N), trabecular separation (Tb. Sp), and trabecular thickness (Tb. Th). Cross-sectional images of the mid-diaphysis of femur were used for 3-dimensional histomorphometric analysis of cortical bones. For cortical bones, the ROI selected for analysis was 10% of femoral length in the mid-diaphysis of the femur to determine cortical bone mineral density (Ct.BMD), cortical thickness (Ct. Th), and cortical porosity (Ct. Po).

### Measurement of bone biomechanics

The biomechanical properties of the bones were determined according to the procedures described by Mattila et al. [22]. The bones were subjected to three-point bending test on a universal testing machine (Instron Corp., CAT NO 2752-005, Canton, MA) until failure. Three-point bending strength was measured with a constant span length of 10 mm. The compressive loading speed in all tests was 0.1 mm/s. The obtained load-time curve was converted into a load-displacement curve. Breaking force was defined as bending load at failure. Stiffness was calculated as the slope of the linear (elastic) part of the load-displacement curve.

### Histological analysis

The right femora were decalcified in 5% EDTA in PBS for 2 weeks, embedded in paraffin after dehydration and coronally cut into 5 μm sections. Then immunohistochemical staining of collagen I and TUNEL staining were performed as described below.

#### Immunohistochemical staining

In brief, bone sections were deparaffinized in xylene and rehydrated in diminishing concentrations of ethanol, followed by subsequent incubation in 3% H_2_O_2_ for 10 min at room temperature to eliminate endogenous peroxidase activity. Antigen-retrieval was performed by heating the sections for 9 min in EDTA buffer (pH 6.0). Thereafter, the slides were blocked with goat serum albumin (BOSTER, Pleasanton, USA) for 15 min at 37 °C, and then incubated overnight at 4 °C with primary antibody specific for Collagen I (Abcam, Cambridge, USA). After rinsing, the slides were incubated for 1 h at 37 °C with secondary antibody of goat anti-rabbit IgG (HRP-labeled, Santa Cruz Biotech). After rinsing again, the sections were stained with DAB. Finally, the sections were rinsed in distilled water, and counterstained with haematoxylin. Finally, the area fraction of collagen I were developed and quantified by Image J version 1.6 (National institutes of Health, USA).

#### TUNEL staining

TUNEL assay was performed with an in situ cell death detection kit (G3250, Promega, USA) according to the manufacturer’s protocol. Briefly, paraffin-embedded sections were deparaffinized, washed with PBS and pretreated with proteinase K (20 μg/ml) for 15 min. The equilibration buffer was applied directly to each section and incubated for 10 s. Sections were then incubated with rTdT enzyme in a humidified chamber at 37 °C for 1 h. After being labeled, sections were washed and mounted under coverslips. DNA fragmentation was detected by selecting four fields at 100× magnification in each section. All images were analyzed by Adobe Photoshop CS2 to quantify the signals. Data were expressed as the number of apoptotic cells per high-power field.

### Western blot

Bone proteins were extracted from the samples after mechanical testing. After washing medullary cavity, the bones were ground into powder in liquid nitrogen, homogenized directly in RIPA buffer supplemented with Na_3_VO_4_, NaF, PMSF and Cocktail, and then centrifuged at 4 °C for 10 min at 12,000×g. The resulting supernatant was separated by SDS-PAGE and transferred to membranes. After blocking, primary antibodies of anti-osteocalcin (Santa Cruz Biotechnology, USA) anti-collagen I (Abcam, Cambridge, USA) and anti-β-actin (Santa Cruz Biotechnology, USA) were added and incubated overnight at 4 °C. Then, the membranes were incubated with secondary antibody of goat anti-rabbit and anti-mouse IgG (HRP-labeled, Santa Cruz Biotech) in PBST for 40 min. Finally, specific bands were developed and quantified by Image J version 1.6 (National institutes of Health, USA.

### Cell line and cell culture

Murine MC3T3-E1 pre-osteoblast cell line was were grown in α-minimum essential medium (α-MEM) (Gibco BRL, Grand Island, NY, USA) with 10% fetal bovine serum and 1% penicillin–streptomycin (Gibco BRL) under 5% CO_2_ atmosphere at 37 °C.

### Cytotoxicity test

To analyze the cytotoxicity of fenofibrate on MC3T3 cells, a CCK-8 assay (Chengjian, NanJing, China) was used according to the manufacturer’s instructions. Briefly, MC3T3 cells were plated in 96-well plates at a density of 5 × 10^3^/well. And then, solutions containing various concentrations of fenofibrate (0, 25, 100, 200 ng/μL) were added. The standard protocol for assessing cell viability was then carried out.

### Quantitative PCR

MC3T3 cells were plated in 12-well plates at a density of 1.8 × 10^5^/ml. After incubation for 12 h, total RNA was extracted using TRIzol (Takara, Shiga, Japan). The cDNA was then synthesized from total RNA by a reverse transcriptase cDNA synthesis kit (Takara, Shiga, Japan). Quantitative PCR was carried out on a 7500 Real-Time PCR System (Applied Biosystems, Waltham, MA, USA) using SYBR Premix Ex Taq (Takara, Shiga, Japan). Forward and reverse primers were listed in Table 1. All data were normalized to β-actin and assays were performed in triplicate.

**Table 1:** Primer sequences used for quantitative PCR

Primer	Forward (5′–3′)	Reverse (5′–3′)
Runx2	AACGATCTGAGATTTGTGGGC	CCTGCGTGGGATTTCTTGGTT
Osteocalcin	CTGACCTCACAGATCCCAAGC	TGGTCTGATAGCTCGTCACAAG
Spp1	AGCAAGAAACTCTTCCAAGCAA	GTGAGATTCGTCAGATTCATCCG
Sp7	ATGGCGTCCTCTCTGCTTG	TGAAAGGTCAGCGTATGGCTT
Bcl2	GTCGCTACCGTCGTGACTTC	CAGACATGCACCTACCCAGC
Caspase3	ATGGAGAACAACAAAACCTCAGT	TTGCTCCCATGTATGGTCTTTAC
β-actin	CATGTACGTTGCTATCCAGGC	CTCCTTAATGTCACGCACGAT

### Statistical analysis

The data were expressed as mean ± standard deviation (SD). Student’s *t*-test and one-way ANOVA were used to assess the significance of differences using GraphPad Prism version 4.0. The post hoc analysis was also carried out. A *P*-value less than 0.05 was considered statistically significant.

## Results

### Fenofibrate causes a further decrease in bone mass of the HFD-induced T2DM mice

**Fig. 1:**
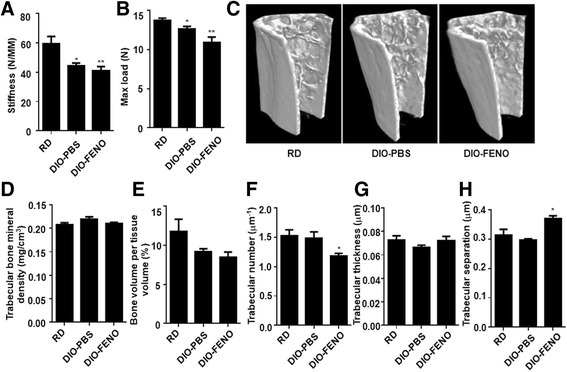
HFD-induced T2DM mice treated with fenofibrate showed low bone quality. Tissue-level mechanical properties, including **a** bending stiffness and **b** maximum load, of the femur mid-shaft were measured by three-point bending test in RD, DIO-PBS and DIO-FENO mice. **c** Representative images of micro-CT analysis of the mice femur microstructure. **d** Trabecular BMD, **e** BV/TV, **f** trabecular number, **g** trabecular thickness and **h** trabecular separation of the bones of HFD-induced T2DM mice were measured by the Micro-CT (*n* = 7). ^*^ *P* < 0.05, ^**^ *P* < 0.01, ^***^ *P* < 0.001

To determine the changes in bone biomechanics after fenofibrate treatment in T2DM mice, the three-point stress bending test and micro-CT analysis were performed, respectively. As shown in Fig. 1a and b, the femoral stiffness and the max load of the DIO-PBS group decreased significantly compared with the RD group (F (2, 12) = 9.061, *P* = 0.004 for Fig. 1a; and F(2,13) = 10.22, *P* = 0.002 for Fig. 1b). The max load value of the DIO-FENO group was significantly decrease compared to the DIO-PBS group (*P* < 0.05), while there was no significant difference in the bone stiffness values between these two groups.

In order to clarify the causes of reduced bone biomechanical properties after treatment with fenofibrate, micro-CT analysis was performed. As shown in Fig. 1c, the trabecular bone mass in DIO-FENO group was lower than that in the DIO-PBS group. There were no significant differences in trabecular bone related parameters such as BMD, BV/TV, trabecular bone numbers, trabecular bone thickness, and trabecular bone separation between the DIO-PBS group and RD group. (Fig. 1d-h). This indicates that the bone quality decreased in the HFD-induced T2DM (DIO-PBS) mice, which means that the biomechanical properties of T2DM bones significantly decreased without bone mass change. It suggests that HFD-induced T2DM causes a change in bone hardness, leading to bone brittleness increase. Compared with RD group or DIO-PBS group, the trabecular number (Fig. 1f) (F (2,13) =5.113, *P* = 0.023) in the DIO-FENO group significantly decreased whereas the trabecular separation (Fig. 1h) (F (2,14) = 11.71, *P* = 0.001) in the DIO-FENO group significantly increased (*P* < 0.05).

These results indicate that trabecular bone mass decreases in the T2DM mice after fenofibrate treatment, and thus leads to a decrease in bone biomechanics.

### Fenofibrate causes a decrease in bone porosity of the HFD-induced T2DM mice

**Fig. 2:**
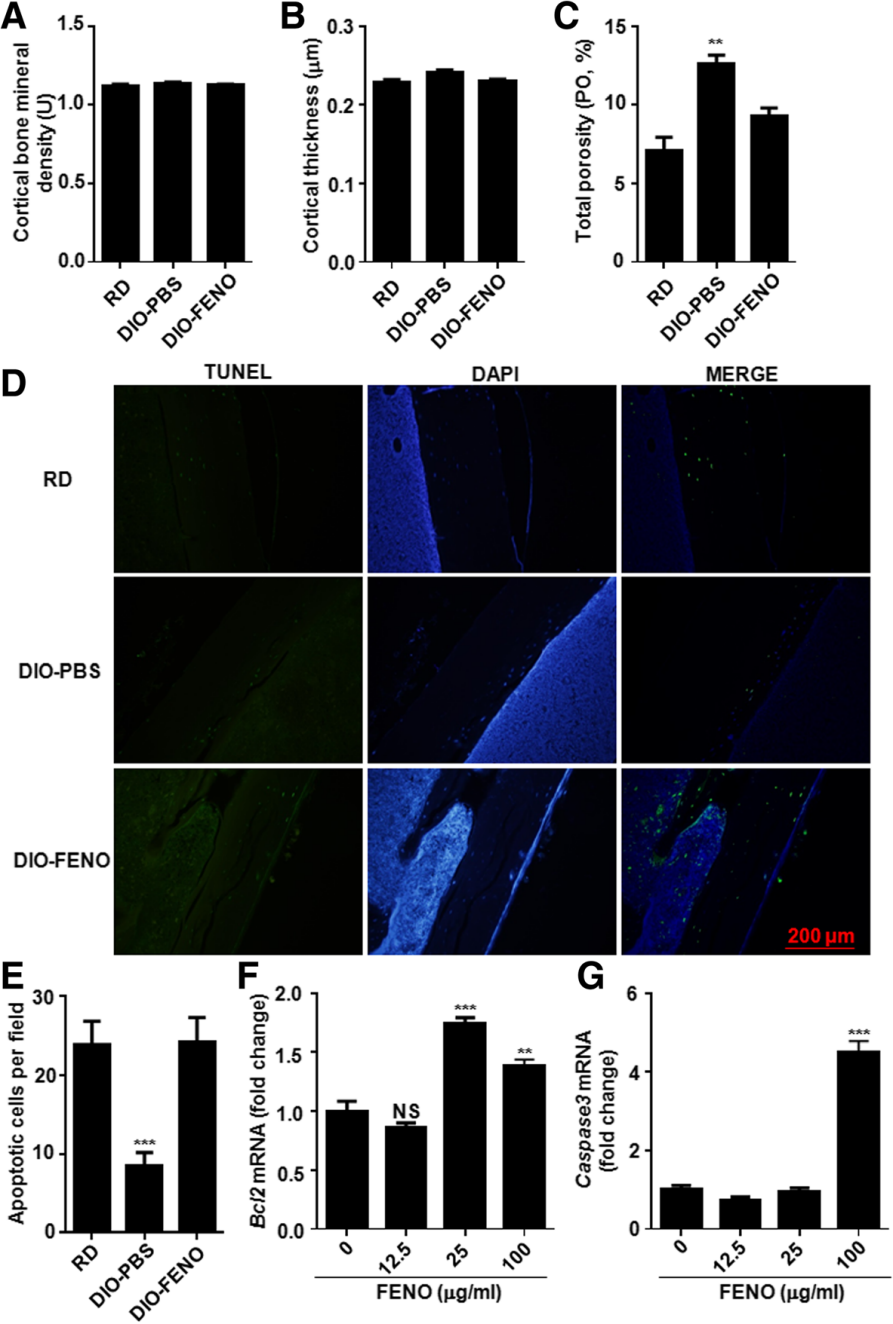
HFD-induced T2DM mice treated with fenofibrate showed low cortical porosity. Analysis of **a** cortical bone mineral density, **b** cortical thickness, and **c** cortical porosity of femur (*n* = 7). **d** Representative sections of femur from different groups were stained with TUNEL, and **e** apoptotic cells per field were counted from 5 slides (10 fields in each slide). **f** Bcl2 and **g** caspase 3 expression in MC3T3 cells treated with fenofibrate at different concentrations detected by qPCR. Results are presented as mean ± SD obtained from three repeated measurements. ^*^ *P* < 0.05, ^**^ *P* < 0.01, ^***^ *P* < 0.001

Bone fragility is significantly increased in diabetic patients, and the porosity of cortical bones has a significant impact on brittle fractures [23]. To evaluate the microstructure of cortical bones from different groups, micro-CT was performed. There were no significant differences in cortical bone BMD and thickness (Fig. 2a and b) among the three groups. However, the cortical bone porosity in the DIO-PBS group was significantly increased (*P* < 0.05) (Fig. 2c) (F (2,10) =17.01, *P* = 0.0006). After fenofibrate treatment, the cortical bone porosity in the DIO-PBS group was significantly decreased than the DIO-PBS group (Fig. 2c). No significant difference was found between the RD group and the DIO-PBS group. This indicates that the cortical porosity of HFD-induced T2DM mice was increased, which might cause the decrease of bone biomechanical properties. However, a contrary result showed that the cortical porosity was decreased after fenofibrate treatment compared with that of DIO-PBS group.

To further explore the mechanism of the contrary result in DIO-FENO group, TUNEL staining was performed. Cell apoptosis in the cortical bones of the DIO-FENO group significantly increased after treated with fenofibrate (Fig. 2d and e). At the same time, the expression levels of Bcl2 and caspase 3 mRNA were significantly increased in the MC3T3 cells after treated with 100 μg/mL fenofibrate (Fig. 2f and g). Therefore, we infer that fenofibrate might promote apoptosis of osteoblasts, resulting in a decrease in bone porosity.

### Fenofibrate causes a significant decrease in the expression of collagen I and osteocalcin in the HFD-induced T2DM mice

**Fig. 3:**
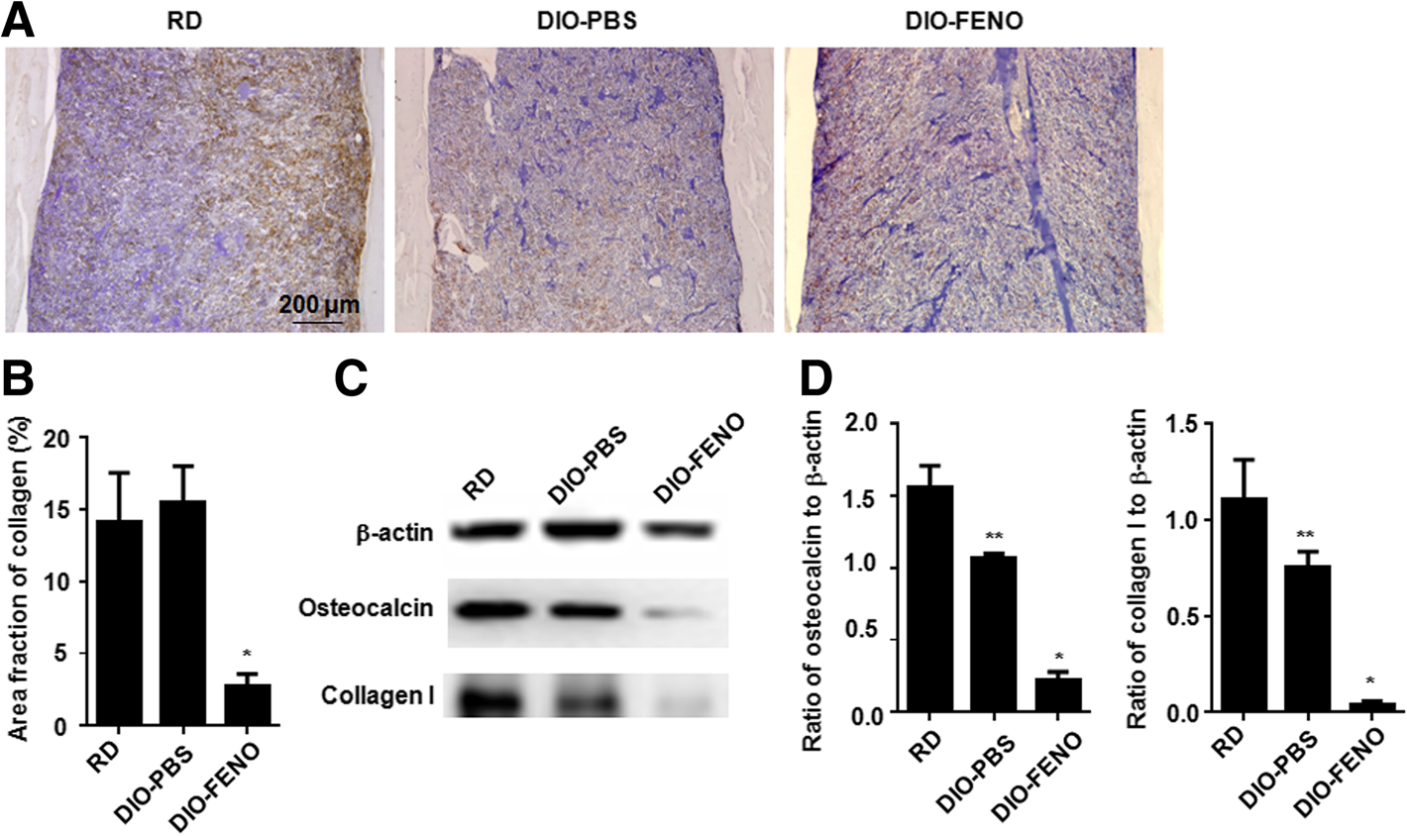
HFD-induced T2DM mice treated with fenofibrate down regulated the expression of osteoblast extracellular matrix. **a** Immunohistochemical staining for Collagen I in representative sections of femur from different groups. Magnification was 100×. **b** Area fraction of collagen I was counted from 5 slides (10 fields in each slide). **c**, **d** Western blot analysis for Collagen I and osteocalcin protein expression extracted from the bones of different groups. β-actin was used as control. ^*^ *P* < 0.05, ^**^ *P* < 0.01, ^***^ *P* < 0.001

In order to clarify the changes in extracellular matrix composition in the bones of HFD-induced T2DM mice, immunohistochemical staining was performed to evaluate the expression level of collagen I. The results showed that the expression of collagen I in DIO-PBS group was obvious lower than that in the RD group, while that in the DIO-FENO group was further reduced (Fig. 3a). Statistically, the expression of collagen I in the DIO-FENO group was significantly decreased than that in the RD group and the DIO-PBS group (*P* < 0.05) (Fig. 3b). Meanwhile, Western blot also found that the protein expression of collagen I and osteocalcin in the DIO-FENO group was reduced (Fig. 3c, d). Therefore, fenofibrate may decrease the expression of collagen I and osteocalcin in osteoblasts extracellular matrix, thus affecting the bone structure and strength.

### High dose of fenofibrate may affect osteoblast activity by down-regulating Runx2

**Fig. 4:**
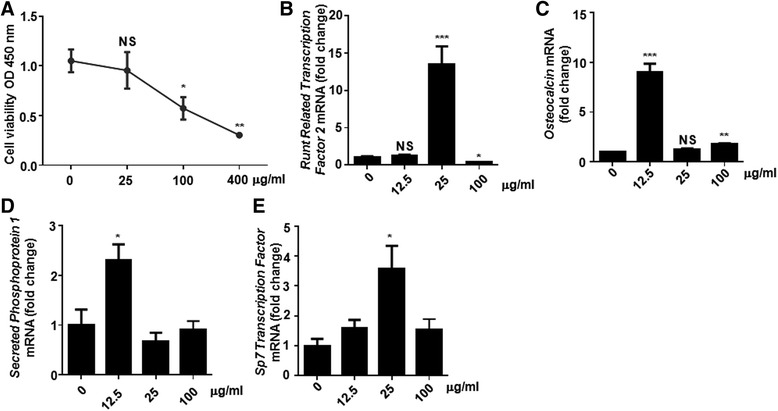
The effect of high dose fenofibrate on osteoblast viability and its regulation on the expression of Runx2 gene. **a** Fenofibrate decreased the MC3T3 cell viability at different concentrations measured by a CCK-8 assay. The expression of **b** Runx2, **c** osteocalcin, **d** Spp1 and **e** Sp7 in MC3T3 cells after treated with different concentrations of fenofibrate was analyzed by qPCR. Data are expressed as fold changes compared to the untreated control, and were presented as mean ± SD. ^*^ *P* < 0.05, ^**^ *P* < 0.01, ^***^ *P* < 0.001

To investigate how fenofibrate affects the expression of collagen I and osteocalcin in T2DM mice, in vitro experiments were performed using MC3T3 cells. CCK-8 assay results showed that the cell viability was not significantly changed at the low concentration of fenofibrate (0 and 25 μg/ml) (Fig. 4a). At the low concentration of 12.5 μg/ml, the osteocalcin and Spp1 genes were significantly increased, while at 25 μg/mL, the Runx2 and Sp7 genes were increased greatly (*P* < 0.05, Fig. 4b-e). These results were similar to the finding that fenofibrate may regulate the differentiation of osteoblast through bone morphogenetic proteins [24]. However, this study was focused on the side effects of fenofibrate on the bones of T2DM mice with dyslipidemia. Thus, the protective mechanism of fenofibrate at low concentrations was not discussed deeply. Moreover, with the increase of concentration, the cell viability was decreased greatly at the high concentrations of fenofibrate (Fig. 4a). The expression of osteoblast markers was also significantly decreased. At the high concentration of 100 μg/ml, the Runx2 mRNA levels were decreased greatly compared with the untreated cells (*P* < 0.05) (Fig. 4b). And Osteocalcin, Spp1, Spp7 were also decreased compared with the low concentrations of fenofibrate group (Fig. 4c-e). Therefore, it is concluded that high dose of fenofibrate may inhibit the expression of Runx2 gene in osteoblasts, and thus have a negative effect on the bones of T2DM mice with dyslipidemia.

## Discussion

T2DM is sometimes associated with skeletal system complications characterized by reduced bone mass and increased bone fragility [1, 2, 23]. In this study, we used HFD-induced diabetic mouse model to investigate the effects of fenofibrate, a lipid-lowering drug, on skeletal system during the treatment of diabetic dyslipidemia. It was found that fenofibrate could further reduce the biomechanical parameters of T2DM mice by modulating the expression of Runx2, thereby increasing the risk of diabetic brittle fracture.

Brittle fracture is a common complication in T2DM patients, which is characterized by a normal or slightly increased BMD value and an increased risk of fracture [25]. In the HFD-induced T2DM mice, we found no significant difference in BMD between the DIO-PBS group and the RD group, while the biomechanical activity was significantly reduced, thus inducing skeletal fragile fracture. However, the mechanism of brittle fracture of T2DM is not entirely clear. The Health, aging, and body composition (HABC) research found that T2DM patients had increased serum levels of cystatin C, an index of impaired renal function, which might lead to vitamin D uptake disorder [26]. In this research, the osteocalcin expression in the bone tissues of DIO-PBS group and DIO-FENO group was significantly lower than that in the normal group. Diaz-Lopez et al. also reported a significant reduction in both uncarboxylated and carboxylated osteocalcin in the sera of patients with T2DM [27]. Decreased osteocalcin levels indicate that osteoblast activity in T2DM patients is inhibited [28].

At present, metformin, pioglitazone and other hypoglycemic agents are the clinically preferred drugs for treatment of T2DM [1, 13, 29, 30], however, a long-term and high-dose usage can also cause some side effects. DeFronzo et al. found that long-term use of metformin may lead to severe metformin-associated lactic acidosis in some patients, and may further damage their renal function [31]. Stunes et al. found that the use of pioglitazone may activate Peroxisome proliferator-activated receptor-γ (PPARγ), aggravating the loss of bone mass in patients with T2DM [32]. In addition to increased blood glucose in patients with T2DM, dyslipidemia is also the cause of increased mortality in patients [13].

Fenofibrate is the commonly used drug for the treatment of dyslipidemia. FIELD study showed that fenofibrate can effectively reduce blood TG levels and reduce the risk of total cardiovascular events in T2DM patients [17]. Meanwhile, it can delay retinopathy [19], and inhibit hypertrophy of fat cells to alleviate insulin resistance [33, 34]. However, the usage of fenofibrate also causes some side effects like heartburn in primary biliary cirrhosis.

In this study, the number of trabecular bone was significantly reduced after fenofibrate intragastric treatment, and the max load was also decreased significantly. This indicates that the bone loss of T2DM mice is increased after taking fenofibrate. Fenofibrate is an important PPARα agonist [34], and PPARα is mainly involved in the body fat metabolism process, such as fatty acid uptake through cell membranes, fatty acid binding in cells, fatty acid oxidation and lipoprotein assembly and transport [35]. There are also related reports about the impact of PPARα on bones. Stunes et al. found that fenofibrate can significantly alleviate the symptoms of osteoporosis, while pioglitazone increased bone loss and negatively regulated bone structure in the ovariectomized rat model [32]. The different result after lipid-lowering drug treatment may depend on the duration of drug usage and the health of the bones [36]. However, in the HFD-induced T2DM model of this study, the expression of collagen I and osteocalcin in bone tissues was significantly decreased after fenofibrate treatment. High concentration of fenofibrate inhibited the expression of Runx2 and thus inhibited collagen I expression. These results are consistent with the study by Ortuño et al. that Runx2 can promote collagen I expression [37].

Burghardt et al. found that the cortical bone porosity of postmenopausal T2DM patients increased significantly [38], which may be one of the main reasons leading to the increase of bone brittleness. However, the mechanism of increased cortical bone porosity is still unclear. Lotinun et al. found that continuous subcutaneous injection of parathyroid hormone (PTH) can lead to increased cortical bone porosity [39], while Jilka et al. found increased femur trabecular bone mass together with significant increase in cortical bone porosity after knocking out the apoptosis-related genes, such as Bax and Bak, in mice [40]. This study confirmed that cell apoptosis in the cortical bones of T2DM mice was significantly lower than that of the RD group, and cell apoptosis increased significantly after fenofibrate treatment. This result is similar to the report by Li et al. that the apoptosis of triple-negative breast cancer cells increased after the addition of PPARα agonist fenofibrate [41]. We also found that the expression of Bcl2 and caspase3 in osteoblast cell line MC3T3 significantly increased after treated with fenofibrate, compared to those in the untreated cells. This further confirmed that fenofibrate in different concentrations could promote osteoblast apoptosis and thus alleviate the increase in cortical bone porosity.

## Conclusions

In conclusion, we successfully established T2DM mouse model using HFD. After treatment with fenofibrate, the number of trabecular bone was significantly lower, and the expression of collagen I and osteocalcin in bone tissues was significantly decreased. Although the cortical bone porosity had a certain decline compared with that in the model group, osteoblasts viability and biomarkers were significantly reduced after high-dose fenofibrate treatment. However, this cannot alleviate the reduction of bone strength. The reduced bone quality caused by fenofibrate might be due to the down-regulation of Runx2. This suggests that the skeletal system of diabetic patients should be monitored when using fenofibrate to treat dyslipidemia in the clinic, in order to prevent the development of brittle fractures.

## Abbreviations

BMD: Bone mineral density
BV/TV: Bone volume per tissue volume
DIO: Diet-induced obese
FIELD: Fenofibrate Intervention and Event Lowering in Diabetes
HFD: High-fat-diet
LPL: Lipoprotein lipase
micro-CT: Microcomputed tomography
T2DM: Type 2 diabetes mellitus
TG: Triglyceride
